# The attitude of Tunisian medicine resident toward euthanasia

**DOI:** 10.1192/j.eurpsy.2024.1201

**Published:** 2024-08-27

**Authors:** A. Touiti, C. Ben Said, N. Bram

**Affiliations:** ^1^Forensic Psychiatry Departement, Razi Hospital, La Manouba; ^2^Faculty of Medecine of Tunis, Tunis El Manar University, Tunis, Tunisia

## Abstract

**Introduction:**

Euthanasia is the active deliberate ending of life by another person at the explicit request of a patient who is suffering from an incurable condition deemed unbearable by him or her.young doctors in tunisia might be exposed in their daily practice to a request of (E). In some countries the procedure is regulated by law while in others the issue has not been discussed. Before assessing the public opinion the medical core has to be implicated in the debate about the subject.Within the limits of our knowledge this is the first study on the subject in the countries of North Africa

**Objectives:**

To describe the attitudes of tunisian medicine resident toward euthanasia

**Methods:**

The validated questionnaire of physicians’ Attitudes and opinions on assisted suicide and euthanasia was distributed via mails addresses to 50 tunisian resident. The participation was entirely voluntary and anonymity was guaranteed.

**Results:**

Thirty seven medicine resident participate to the study the response rate was 74%. The average age of participants was 28.2years old.The majority;23 were female and 29 had religious beliefs.The most represented speciality was family medicine with 6 participants.Only 2 of doctors were practicing in Europe.About 8 of young doctors were requested for (E).Tunisian medicine residents are generally supportive of the legalization of euthanasia (29), but many have concerns about their own participation in the procedure.

**Conclusions:**

Ethical and legal complexities surround the topic of euthanasia.It is imperative to deepen our understanding of this practice within the context of the North Africa region,in order to formulate a comprehensive and well-informed policy.

**Disclosure of Interest:**

None Declared

